# Annotations

**Published:** 1893-03-18

**Authors:** 


### Annotations.

More Bacteria.

The facts of bacteriology are being piled up apace. Dr. C. S. Sherrington, of the Brown Institution, contributes a carefully prepared paper on "The Escape of Bacteria with the Secretions," and for the same journal Dr. Lockhart Gillespie, of Edinburgh, has written a learned article on the "Bacteria of the Stomach." We are not prepared to say what results of a practical kind have accrued or may accrue to the daily work of the physician from these papers; neither, indeed, are the authors of the papers themselves. Nevertheless, practising physicians regard with entire approval and satisfaction the unconquerable efforts which are being made on all hands to wrest from Nature the most steadfastly locked of her secrets, and to turn them to practical use in the service of man. According to Dr. Lockhart Gillespie the bacteria which are more or less indigenous to the stomach may be counted by the dozen. The names of some of them are quite familiar, whilst those of others will be strange to many. Whatever use medicine may or may not be able to make of many of the facts of bacteriology, we are at any rate grateful for the facts. With knowledge we may do something, but with ignorance we can do nothing good at all except by chance and good luck. The medical science of the latter end of the nineteenth century shows an activity which has hardly ever been paralleled. The younger members of our profession are among the most energetic and successful of all the pioneers of science. Perhaps we may yet, before the nineties are out, realise some of our most brilliant dreams, and make the miracles of the nineteenth century more wonderful than those of the first.

"A Plague of Specialists."

Sir Dyce Duckworth, who was one of the distinguished guests at the Royal Medical Society's dinner at Edinburgh the other day, gave short shrift to the specialists which overwhelm the present generation. There was, he said, "in London a plague of specialists: the profession was literally degraded by them." The medical profession in London is "degraded" by its specialists. That is what we understand Sir Dyce Duckworth to mean. Is it the fact? And if it be, one would like a few more details. In what way do specialists "degrade" the medical profession? Is it merely by their numbers; or by their pretence of knowledge and capacity which is not justified by results? For our part, we have long felt that the profession is deeply degraded in one particular by its specialists, and that is in its literature. What is the history of many a specialist and his published works? "Go to," says he; "I will drudge no longer: I will become a specialist." What then does he proceed to do? He chooses his subject, and forthwith sets to work to "read up" anything which has been written upon it during the last twenty or thirty years. He never thinks of going further back than that. To the average specialist medicine did not exist before his own subject was made a specialty. He ransacks books, magazines, medical journals, stealing as he goes. He is not clever even as a thief; he steals, without discrimination, bad and good alike. Out of materials so collected he sits down and compiles—what? A book? No! A crude hotch-potch of facts and fancies, sense and nonsense, without method, point, or purpose. Then he hies him to a publisher, and with him makes the best terms he can. The terms are of little moment: the thing to be done is to get so many pages of print within boards, with a publisher's imprimatur, and his own name and degrees in large letters on a conspicuous part of the cover. Nothing now remains but to advertise the book in as public a way as it is safe to indulge in, and to remove to a popular doctor's street at the West-end, and the thing is done. But how can the thing be made to pay? How does the specialist live? for he cannot eat his own compilation, though it lie on his shelves in unsold hundreds. His foolish brethren help him to live. They see in the Medical Directory that he has published a book—say, on "Flatulalgia" or "Aquabrash," and forthwith they send their obstinate cases of "Aquabrash" or "Flatulalgia" to the distinguished author, quite certain that they will come home no better than they went, and will in future be content with the family practitioner. This may be good business from the practitioner's point of view, but let him consider the "degradation" of the profession. Lord Salisbury sees that medicine, in the hands of its best representatives is steadily being raised to honour. We who are in it see that in the hands of its worst it is as steadily being dragged through the mire. Who will suggest an efficient means of checking a superfluous and degrading hyper-specialism?

How to Catch Rheumatism.

The plan is simple, and is invariably successful when diligently pursued. Clothe as warmly as possible, with flannels next the skin, and sealskins outermost from November to the beginning of March. Then on the first clear, sunny day in March, when the wind is in the north or north-east, take off all outer wraps, mantles, capes, sealskins, and the like; wear gowns of a light and thin material, and go for a walk in the park, or other open and unprotected place. Sit down full in the wind on a convenient seat, and sit for half-an-hour. Then go home, and wake with a successful attack of rheumatism next morning. If perchance the rheumatism should fail, it is probable that pleurisy or pneumonia may be the reward. But if, by a miracle, neither the one nor the other repays the trouble taken, then go out and repeat the same tactics the next day, and the next and the next, until success is assured. The recipe is warranted never to fail, if persevered with for a sufficient length of time. A plan almost equally good is the one followed by two young ladies last week. They had both had rheumatic fever previously; yet because the sun happened to shine brightly in at the dining-room window for a few hours, they allowed the fire to go out. They sat without fire the remainder of the day and evening. The following being a bright morning, they did not have the fire lighted at all. They were both extremely surprised when they were attacked by rheumatic pains in all their limbs, and blamed the neighbourhood. One of these lady philosophers was a physician's daughter, and the other a very successful hospital nurse and assistant matron. So true is it that "knowledge comes whilst wisdom lingers." But to a man who takes physiology and climatology seriously such conduct savours of the very insanest of all the forms of insanity.

